# Downregulation of CD200 in the placenta of preeclampsia: a potential regulator of macrophage-mediated immune imbalance at the maternal-fetal interface

**DOI:** 10.3389/fimmu.2026.1830162

**Published:** 2026-06-30

**Authors:** Shuzhen Huang, Zhaowen Yang, Zijian Li, Quanqi Liang, Zheng Xiao, Heng Yang, Yongkun Chen, Suiwen Wen, Kaishu Li, Cui Peng

**Affiliations:** 1 Department of Gynecology, The Affiliated Qingyuan Hospital (Qingyuan People’s Hospital), Guangzhou Medical University, Qingyuan, China; 2 Institute of Digestive Diseases, the Affiliated Qingyuan Hospital (Qingyuan People’s Hospital), Guangzhou Medical University, Qingyuan, China; 3 Department of Neurosurgery, The Affiliated Qingyuan Hospital (Qingyuan People’s Hospital), Guangzhou Medical University, Qingyuan, China

**Keywords:** CD200, macrophages, placenta, preeclampsia, serum biomarkers

## Abstract

**Objective:**

To investigate the role and potential mechanism of CD200 in the pathogenesis of preeclampsia (PE) and to clarify its potential value as a biomarker candidate and possible therapeutic target for PE.

**Methods:**

This retrospective study enrolled 57 patients with PE and 49 women with normal full-term pregnancies. Clinical data were collected, and serum biomarkers were measured. The GSE93839 dataset was utilized to screen for differentially expressed genes in PE placental tissues. Hematoxylin-eosin (HE) staining, immunohistochemistry (IHC), and immunofluorescence (IF) techniques were employed to analyze the expression and localization characteristics of CD200 in placental tissues. Exploratory path analysis was conducted to investigate potential indirect associations between CD200 expression and PE through serum biomarkers.

**Results:**

Bioinformatics analysis identified CD200 as a candidate downregulated gene associated with PE. Immunohistochemistry revealed a significantly lower expression level of CD200 in the placental tissues of PE patients (p < 0.05). Immunofluorescence experiments demonstrated co-localization of CD200 with CD68^+^ macrophages, suggesting a potential association with macrophage-related immune regulation at the maternal-fetal interface. Exploratory path analysis revealed statistically significant indirect associations between CD200 expression and PE through serum biomarkers, including high-density lipoprotein cholesterol (HDL-C), albumin, alanine aminotransferase (ALT), lactate dehydrogenase (LDH), creatine kinase (CK), and creatine kinase-MB (CK-MB) (p < 0.05).

**Conclusion:**

The expression of CD200 is significantly downregulated in the placental tissues of PE patients. This alteration may induce immune imbalance at the maternal-fetal interface and placental dysfunction, and may also be linked to the systemic pathological injury process in PE through its associations with multiple serum biomarkers.

## 1 Introduction

Preeclampsia (PE) is a severe complication characterized by new-onset hypertension after 20 weeks of gestation accompanied by end-organ damage. With a global occurrence rate of 5-8%, it is a leading cause of maternal and perinatal mortality (1). Although the “placental ischemia-systemic inflammation” hypothesis is widely accepted—wherein inadequate trophoblast invasion leads to impaired placental perfusion, followed by the release of inflammatory mediators that induce maternal vascular endothelial dysfunction (2)—the upstream molecular events associated with immune imbalance at the maternal-fetal interface remain poorly understood (3).

Within the molecular network that maintains maternal-fetal immune tolerance, immune checkpoint molecules have garnered significant attention due to their precise regulatory role in myeloid cells. CD200 (also known as MOX1/OX-2) is a transmembrane glycoprotein that delivers potent immunosuppressive signals by binding to CD200R1 on macrophages/dendritic cells, functioning as a critical checkpoint that restrains myeloid cell activation through Dok-1/Dok-2-mediated suppression of Ras/MAPK signaling (4, 5). Furthermore, CD200-CD200R1 signaling promotes M2-like anti-inflammatory macrophage polarization via the CREB-C/EBP-β transcriptional pathway, upregulating Arg1 and TGF-β while suppressing NF-κB-driven pro-inflammatory cytokine production (6). Conversely, CD200 deficiency has been associated with sustained M1-like pro-inflammatory macrophage activation characterized by increased TNF-α and IL-6 production (7). This immunomodulatory mechanism is fundamentally distinct from the heme oxygenase (HO) system — particularly HO-1 (HMOX1) — which functions primarily as an antioxidant enzyme that catalyzes heme degradation into biliverdin, carbon monoxide, and ferrous iron, thereby counteracting oxidative stress at the maternal-fetal interface (8). CD200 is highly expressed in placental villous trophoblasts during early pregnancy, suggesting its physiological necessity in establishing maternal-fetal immune tolerance (9). However, the expression profile of CD200 in PE placentas and its association with macrophage dysfunction have not been elucidated.

Based on the established role of CD200 in modulating macrophage activation and inflammatory responses, we hypothesize that downregulation of CD200 in PE placentas may be associated with impaired CD200-CD200R1-mediated immunoregulatory signaling and altered macrophage-related inflammatory patterns at the maternal-fetal interface. This association may involve reduced CD200-CD200R1 engagement on placental macrophages and/or altered paracrine communication between trophoblasts and adjacent immune cells. The resulting release of inflammatory factors such as TNF-α and IL-6 may contribute to a local inflammatory milieu that is linked to endothelial dysfunction and systemic manifestations of PE. Previous studies have focused on the role of CD200 in tumors and autoimmune diseases (5), while its function in pregnancy-related immune disorders remains unexplored.

This study is the first to integrate clinical cohorts, spatial co-localization techniques, and exploratory path analysis to investigate the association between CD200 downregulation and macrophage-mediated immune imbalance in PE, thereby exploring potential links between local immune alterations and systemic organ injury-related biomarkers. The aims of this study are to systematically characterize the expression profile of CD200 in PE placentas, examine its association with macrophage distribution and polarization markers, and explore potential indirect pathways connecting CD200 downregulation with maternal multi-organ dysfunction.

## 2 Methods

### 2.1 Study cohort and ethical approval

This retrospective study consecutively enrolled 106 pregnant women with singleton pregnancies who underwent delivery at the Affiliated Qingyuan Hospital, Guangzhou Medical University (Qingyuan People’s Hospital) from January 2023 to April 2025. The cohort included 57 patients with PE and 49 women with normal term pregnancies. The age of the participants ranged from 19 to 47 years, with a mean age of 32 years.

The diagnosis of PE strictly adhered to the diagnostic criteria outlined in the *Guidelines for the Diagnosis and Management of Hypertensive Disorders in Pregnancy (2020)* issued by the Hypertensive Disorders in Pregnancy Study Group of the Chinese Society of Obstetrics and Gynecology. The criteria were defined as the onset of systolic blood pressure ≥140 mmHg and/or diastolic blood pressure ≥90 mmHg after 20 weeks of gestation, accompanied by any of the following: 1. random urine protein (++); 2. a urine protein/creatinine ratio ≥0.3; or 3. 24-hour urinary protein quantification ≥0.3 g. The inclusion criteria for the control group were: blood pressure <140/90 mmHg and term pregnancy without obstetric complications or other medical or surgical comorbidities.

The exclusion criteria were: (1) pregnant women under 18 years of age; (2) those with gestational diabetes mellitus; (3) those with primary autoimmune diseases such as systemic lupus erythematosus or Sjögren’s syndrome; and those with a history of chronic hypertension.

Severe PE was defined by the presence of any of the following: systolic blood pressure ≥160 mmHg or diastolic blood pressure ≥110 mmHg, thrombocytopenia, renal insufficiency, impaired liver function, pulmonary edema, or cerebral/visual disturbances. Early-onset PE was defined as PE requiring delivery before 34 weeks of gestation. Fetal growth restriction was defined as an estimated fetal weight <10th percentile for gestational age based on standard growth charts. Cases were identified from prenatal ultrasound records and confirmed at birth by pediatric assessment.

This study was approved by the Ethics Committee of Qingyuan People’s Hospital (Ethics Approval No.: QYPH-2023-032). All participants provided written informed consent prior to sample collection and data analysis. The entire research process strictly followed the ethical principles of the Declaration of Helsinki.

### 2.2 Clinical data collection and biospecimen processing

Clinical baseline data were collected for all participants, including age, body mass index (BMI), systolic blood pressure, diastolic blood pressure, history of miscarriage, gravidity, parity, gestational age at delivery, mode of delivery, neonatal sex, and birth weight.

Fasting peripheral venous blood (5 mL) was collected from all participants before delivery. The samples were centrifuged at 3000×g for 10 minutes at 4 °C to separate the upper serum layer. Serum biomarkers related to metabolism, liver and kidney function, myocardial injury, and inflammation were measured using a Beckman Coulter AU5800 automatic biochemical analyzer.

Placental tissue was collected immediately after fetal delivery. Residual blood was thoroughly removed by rinsing with normal saline. According to the placental quadrant method, a 1 cm×1 cm×1 cm sample of villous tissue was obtained from each quadrant. Simultaneously, a 1 cm segment of umbilical cord tissue from the placental end was collected. All tissue samples were immediately fixed in 4% paraformaldehyde solution for subsequent histopathological examination.

### 2.3 Bioinformatics analysis

The gene expression microarray dataset GSE93839 for PE placental tissue was obtained from the NCBI GEO public database. To minimize false positives, Benjamini-Hochberg FDR correction was applied after differential analysis to generate adjusted p-values for multiple-testing assessment. Variance estimates were stabilized using empirical Bayes moderation (eBayes), and missing values were imputed by KNN prior to testing. Differential expression analysis of genes in invasive cytotrophoblasts and endovascular cytotrophoblasts between the PE group and the normal control group was performed using the R software (version 3.2.3 and 4.4.2) package ‘limma’. The screening thresholds were set as |log2(fold change)| > 1 and raw p < 0.05.

Gene Ontology (GO) functional enrichment analysis of the differentially expressed genes (DEGs) was conducted using the DAVID database (https://davidbioinformatics.nih.gov/) with *Homo sapiens* as the background to elucidate the biological processes and signaling pathways involved. A protein-protein interaction (PPI) network of the DEGs was constructed using the STRING database (https://string-db.org/) with a confidence threshold of 0.4. Candidate genes were screened based on the intersection of DEGs between invasive cytotrophoblasts and endovascular trophoblasts, with a consistent direction of expression change in both cell subsets. CD200 was then selected for downstream validation. These bioinformatics analyses were used as a hypothesis-generating screening step for candidate gene selection. In addition, for the GSE93839 dataset (platform: GPL16686 [HuGene-2_0-st], Affymetrix Human Gene 2.0 ST Array [transcript (gene) version]), only protein-coding genes were included in the analysis. Spearman’s correlation analysis was performed between the CD200 gene and various macrophage markers (including M1 and M2 types).

### 2.4 Histopathological examination of placental tissues

#### 2.4.1 Hematoxylin and eosin staining

Fixed placental tissues were dehydrated through a graded ethanol series, cleared, and embedded in paraffin. Serial sections of 4 μm thickness were prepared. Following routine deparaffinization and rehydration, HE staining was performed. The sections were mounted with neutral balsam and observed under a light microscope to examine the morphology, structure, and pathological alterations of placental villi. Structural differences in placental tissues between the two groups were assessed. All placental tissue from pregnant women was stained using the HE method.

#### 2.4.2 Immunohistochemistry

A total of 20 placental biopsy samples from women with pre-eclampsia and 20 from healthy pregnant women were used. After deparaffinization and rehydration, paraffin sections were subjected to antigen retrieval using sodium citrate antigen retrieval solution, followed by inactivation of endogenous peroxidase with 3% hydrogen peroxide and blocking with 10% goat serum. The sections were then incubated overnight at 4 °C with a primary antibody against CD200 (1:500, Abmart, China, #TU721691). After rewarming, the sections were incubated with a secondary antibody (Goat Anti-Rabbit/Mouse IgG/HRP Polymer, ZSGB-BIO, China, PV-6000) at room temperature, followed by chromogenic development using DAB (ZSGB-BIO, China, ZLI-9017) and hematoxylin counterstaining. After dehydration and clearing, the sections were mounted. Images were captured using a light microscope. Quantitative analysis of the staining results was performed using ImageJ software. For visual comparison of immunohistochemical staining, the positively stained area and optical density (OD), reflecting the protein expression range and tissue staining intensity, respectively, were quantified within identical regions of interest using ImageJ software. Specifically, the integrated density (IntDen) and the percentage of positively stained area (% Area) were measured for each image. These two parameters were normalized using Z-scores, and their mean value was calculated to construct a comprehensive CD200 expression index.

#### 2.4.3 Immunofluorescence

Placental tissue samples from five women with pre-eclampsia and five healthy pregnant women were used in the IF assay. Paraffin sections were deparaffinized, rehydrated, and subjected to antigen retrieval. After serum blocking, the sections were incubated overnight at 4 °C with a mixture of primary antibodies against CD200 (1:150, Abmart, China, #TU721691) and the macrophage markers CD68 (1:500, Proteintech, China, #66231-2-Ig), iNOS (1:200, SCB, Canada, #SC-7271), and CD163 (1:100, Abmart, China, #MT13803). Following rewarming, the sections were incubated with the following fluorescent secondary antibodies: Alexa Fluor^®^ 488-conjugated AffiniPure F(ab’)_2_ Fragment Goat Anti-Mouse IgG (H+L) (1:500, Cell Signaling Technology, #4408, Danvers, MA, USA) and Alexa Fluor^®^ 555-conjugated AffiniPure F(ab’)_2_ Fragment Goat Anti-Rabbit IgG (H+L) (1:500, Cell Signaling Technology, #4413, Danvers, MA, USA). All incubations were performed at room temperature in the dark. Nuclei were stained with DAPI (1:1000, Abcam, ab104139, Cambridge, UK), and the sections were mounted with an anti-fade mounting medium. Fluorescent signals were acquired using a Zeiss LSM 900 laser scanning confocal microscope (Carl Zeiss, Jena, Germany). Quantitative analysis was performed using Image-Pro Plus software (version 6.0, Media Cybernetics, Rockville, MD, USA). Fluorescent particles were segmented using the Count/Size function with manual color selection, while background and nonspecific signals were excluded. Based on predefined thresholds for pixel intensity and particle size, the numbers of DAPI-stained nuclei and protein-positive particles were quantified. Protein-positive particle counts were then normalized to the corresponding number of DAPI-stained nuclei in each field to account for differences in cell density across images. In addition, the proportions of CD68^+^ CD200^+^, iNOS^+^CD200^+^, and CD163^+^ CD200^+^ double-positive cells were measured using ImageJ (National Institutes of Health, Bethesda, MD, USA) to characterize CD200 expression and localization within placental macrophages. To further delineate the spatial relationship between CD68 and CD200, line-scan analysis was conducted in ImageJ. A transect line was drawn across regions of interest, and fluorescence intensity-distance profiles were generated using the Analyze-Plot Profile function; the distances between signal peaks were subsequently quantified.

### 2.5 Exploratory path analysis

To explore potential indirect associations between placental CD200 expression and PE, we performed a regression-based path analysis using the PROCESS macro in R software (version 4.4.2). Given the cross-sectional design (all variables measured at delivery), this analysis is hypothesis-generating and does not establish causality.

CD200 expression (Z-score composite) was specified as the independent variable (X), PE status as the binary dependent variable (Y), and 12 serum biomarkers as candidate mediators (M). The a-paths (X → M) were estimated using linear regression, and the b-paths (M → Y, adjusting for X) and direct effect (c’-path) were estimated using logistic regression. All path coefficients (a, b, c, c’) were adjusted for maternal BMI, systolic/diastolic blood pressure, gestational age at delivery, mode of delivery, and neonatal birth weight. The indirect effect (a × b) was tested using 5,000 bootstrap resamples; statistical significance was defined as a 95% bias-corrected CI excluding zero. To account for multiple testing, Benjamini-Hochberg FDR correction was applied (FDR-adjusted p < 0.05).

### 2.6 Statistical analysis

Statistical analyses were performed using SPSS software (version 26.0), R software (version 4.4.2), and GraphPad Prism software (version 8.0). The normality of continuous data was assessed using the Kolmogorov-Smirnov (K-S) test. Normally distributed continuous data are presented as mean ± standard deviation ( $\bar{x}$ ± s), and comparisons between groups were conducted using the independent samples t-test. Non-normally distributed continuous data are presented as median (interquartile range) M (P25, P75), and comparisons between groups were performed using the Mann-Whitney U test. Categorical data are presented as number (percentage) n (%), and comparisons between groups were performed using Pearson’s χ² test; Fisher’s exact test was employed when the theoretical frequency was less than 5.

All statistical tests were two-sided, with a significance level (α) set at 0.05. A P-value < 0.05 was considered statistically significant. The significance markers were uniformly defined as follows: *p < 0.05, **p < 0.01, ***p < 0.001, ****p < 0.0001.

## 3 Results

### 3.1 Baseline characteristics of participants with PE and normal term pregnancy

This study cohort comprised a total of 106 participants, including 57 patients with PE and 49 women with a normal term pregnancy. The clinical characteristics of all participants are presented in **Table 1**. Statistical analysis revealed significant differences (p < 0.001) between the PE group and the normal term pregnancy group in BMI, blood pressure (systolic and diastolic), gestational age at delivery, mode of delivery, and neonatal birth weight. Specifically, the PE group exhibited significantly higher BMI, systolic blood pressure, and diastolic blood pressure compared to the normal pregnancy group (all p < 0.001), indicating a marked hypertensive profile. The median gestational age at delivery was significantly lower in the PE group than in the normal term pregnancy group (p < 0.001), demonstrating that delivery occurred substantially earlier in patients with PE, which is consistent with the clinical observation that this condition often necessitates iatrogenic preterm birth. Notably, neonatal birth weight was significantly lower in the PE group compared to the normal pregnancy group (p < 0.001), which is associated with placental hypoperfusion and the early termination of pregnancy affecting fetal growth. Finally, a significant difference was observed in the ratio of vaginal delivery to cesarean section between the two groups (p < 0.001), suggesting that the onset of PE may reduce the likelihood of vaginal delivery while increasing the probability of cesarean section. In contrast, no statistically significant differences were found between the groups regarding maternal age, number of miscarriages, gravidity and parity, maternal type, or neonatal sex (p = 0.462, p = 0.901, p = 0.595, p = 0.940, p = 0.244, and p = 0.136, respectively). In addition, PE-related perinatal management and fetal complications are summarized in **Supplementary Table 1**. Specifically, antenatal magnesium sulfate was administered in 30 cases (53%) and not administered in 27 cases (47%); antenatal corticosteroids were administered in 17 cases (30%) and not administered in 40 cases (70%). Regarding overall disease characteristics, 40 cases (70%) were classified as severe preeclampsia. Furthermore, fetal growth restriction was observed in 30 cases (53%) and absent in 27 cases (47%).

**Table 1:** General clinical characteristics of participants with PE and normal term pregnancy.

General physical signs	Normal group (*n* = 49)	PE group (*n* = 57)	*p*
Age (years)	31.49 ± 4.63	32.28 ± 6.35	0.462
BMI (kg/m²)	21.12 ± 2.75	23.94 ± 3.65	<0.001
Systolic blood pressure (mmHg)	119.00 ± 10.22	155.54 ± 17.74	<0.001
Diastolic blood pressure (mmHg)	77.69 ± 7.86	100.91 ± 11.47	<0.001
Number of abortions	0 (0, 1)	0 (0, 1)	0.901
Number of pregnancies	2 (1, 3)	2 (1, 4)	0.595
Number of deliveries	1 (0, 1)	0 (0, 2)	0.940
Gestational age at delivery (weeks)	39.3 (38.75, 40)	36.3 (34.25, 38.35)	< 0.001
Mode of delivery			< 0.001
Vaginal delivery	45 (91.80%)	12 (21.10%)	
Cesarean section	4 (8.20%)	45 (78.90%)	
Parity			0.244
Primipara	19 (38.80%)	27 (47.40%)	
Multipara	30 (61.20%)	30 (52.60%)	
Neonatal sex			0.136
Male	23 (46.94%)	35 (61.40%)	
Female	26 (53.06%)	22 (38.60%)	
Neonatal birth weight (g)	3207.76 ± 313.03	2386.65 ± 905.33	< 0.001

### 3.2 Analysis of serum biomarker characteristics in patients with PE

#### 3.2.1 Serum metabolic biomarkers in PE

As shown in **Table 2**, significant differences were observed between the PE group and the normal pregnancy group in levels of triglycerides (TG), high-density lipoprotein cholesterol (HDL-C), uric acid, and albumin (all p < 0.05). Specifically, the levels of TG and uric acid were significantly higher in the PE group compared to the control group (p = 0.023; p < 0.001, respectively), whereas the mean values of HDL-C and albumin were significantly lower (p = 0.004; p < 0.001, respectively). These findings suggest that patients with PE may exhibit an atherogenic lipid profile (characterized by hyperlipidemia accompanied by a reduction in protective lipoproteins), along with hyperuricemia and hypoalbuminemia. However, no significant differences were found between the two groups for some conventional metabolic parameters, such as total cholesterol and low-density lipoprotein cholesterol (LDL-C) (p = 0.478; p = 0.114, respectively), indicating a lack of clear association with the pathogenesis of PE.

**Table 2:** Differential analysis of metabolic parameters between pe and normal term pregnancy.

Variable	Normal group	PE group	p
	(n = 49)	(n = 57)	
TC (mmol/L)	6.17 (5.47, 7.13)	6.18 (5.05, 6.94)	0.478
TG (mmol/L)	3.10 (2.46, 3.85)	3.98 (2.58, 5.52)	0.023
HDL-C (mmol/L)	1.85 ± 0.37	1.63 ± 0.36	0.004
LDL-C (mmol/L)	3.38 (2.69, 4.16)	2.97 (2.37, 3.84)	0.113
Uric Acid (μmol/L)	355.98 (283.79, 428.17)	443.56 (330.27, 556.85)	< 0.001
Albumin (g/L)	36.6 (35.40, 38.05)	35.1 (32.35, 36.4)	< 0.001

#### 3.2.2 Serum organ injury markers in PE

Multiple parameters related to renal, hepatic, and cardiac function showed statistically significant differences between the PE group and the normal term pregnancy group (all p < 0.05) (**Table 3**). The median levels of creatinine and urea nitrogen in the PE group were significantly higher than those in the normal group (p < 0.001; p < 0.001, respectively). The median levels of alanine aminotransferase (ALT), aspartate aminotransferase (AST), and gamma-glutamyl transferase (GGT) in the PE group were all elevated compared with the normal group (p = 0.002; p < 0.001; p = 0.018, respectively). Concurrently, the median levels of creatine kinase (CK) and creatine kinase-MB (CK-MB) were higher in the PE group than in the normal group (p = 0.032; p < 0.001, respectively). These results suggest that patients with PE may exhibit significant renal and hepatic impairment, accompanied by myocardial involvement.

**Table 3:** Differential analysis of hepatic/renal function and cardiac injury indicators between PE and normal term pregnancy.

Variable	Normal group (n = 49)	PE group (n = 57)	p
Creatinine	39.9 (37.05, 48.35)	52.7 (44.15, 63.40)	< 0.001
Urea	2.95 (2.72, 3.57)	4.17 (3.17, 5.26)	< 0.001
ALT	9 (7.01, 13.12)	13 (9.51, 18.21)	0.002
AST	16 (14.09, 19.36)	19 (16.52, 24.44)	< 0.001
GGT	8 (6.03, 11.5)	10 (7.54, 15.5)	0.018
CK	64.47 (46.10, 86.74)	77.40 (57.91, 99.53)	0.032
CK-MB	13 (10.07, 14.54)	15 (13.16, 19.58)	< 0.001

#### 3.2.3 Serum inflammatory and immune markers in PE

The results indicated that only the median level of lactate dehydrogenase (LDH) in the PE group was higher than that in the normal pregnancy group (p < 0.001), suggesting a potential association with the pathogenesis of PE (**Table 4**). However, no statistically significant differences were observed between the groups for leukocyte count, platelet count, or D-dimer levels (p = 0.168, p = 0.575, and p = 0.864, respectively), indicating that these parameters may not be significantly associated with the development of PE.

**Table 4:** differential analysis of inflammation-related parameters between PE and normal term pregnancy.

Variables	Normal group (n = 49)	PE group (n = 57)	p
White Blood Cells	9.35 (8.25, 10.98)	10.1 (8.70, 12.40)	0.168
Platelets	242.10 ± 58.96	234.56 ± 76.26	0.575
D-dimer	1.88 (1.35, 3.00)	1.88 (1.49, 2.77)	0.864
LDH	179 (155.5, 207.5)	209 (178, 251)	< 0.001

### 3.3 Validation of CD200 expression through bioinformatics and experimental approaches

#### 3.3.1 Bioinformatics analysis of CD200 expression in PE

**Figure 1:**
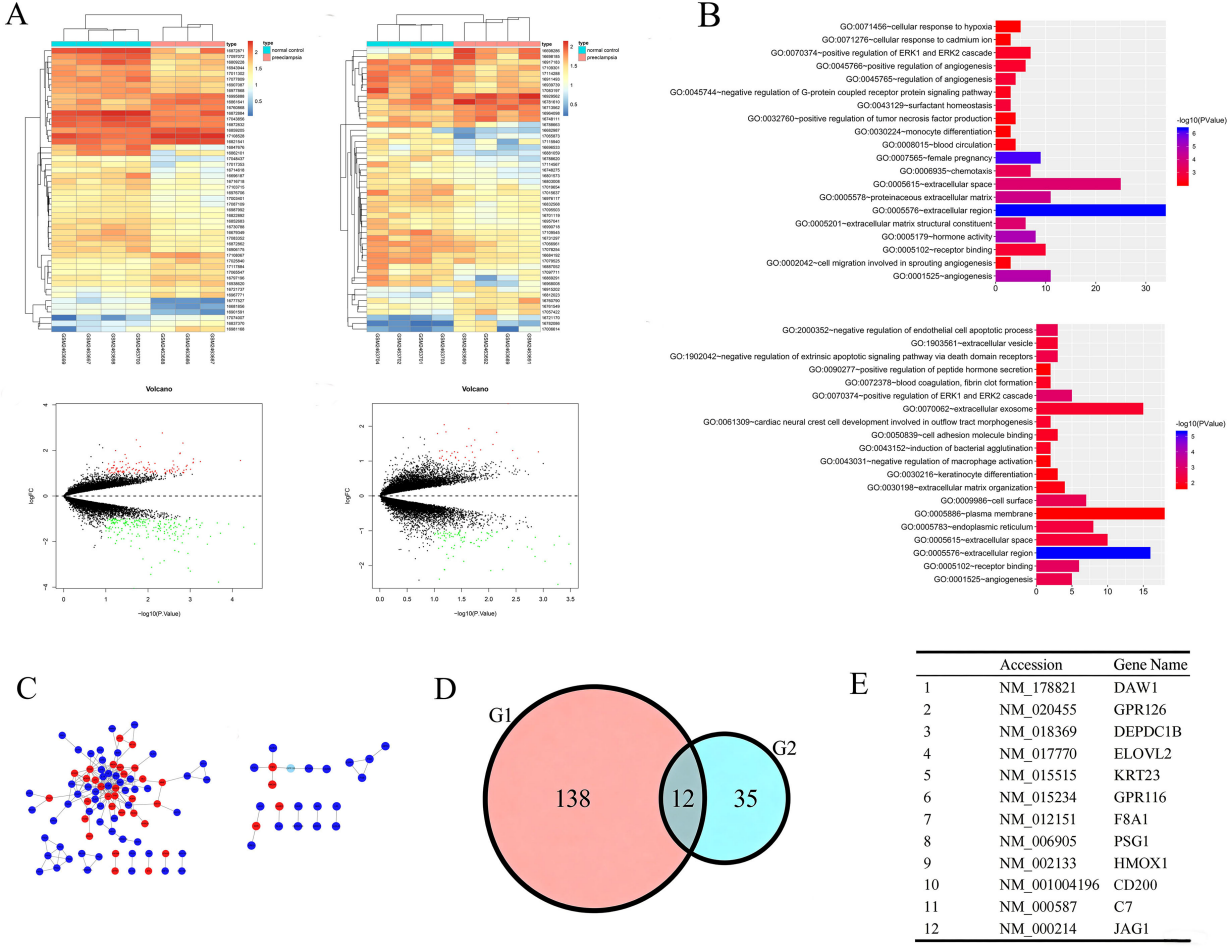
Gene expression profiles were analyzed by several bioinformatics methods based on the GEO database. **(A)** Clustered heatmaps and volcano plots of the top 50 differentially expressed genes in invasive trophoblasts (left) and endovascular trophoblasts (right). **(B)** Functional enrichment analysis of differentially expressed genes. **(C)** Protein-protein interaction (PPI) network analysis of differentially expressed genes in invasive cytotrophoblasts (left) and endovascular trophoblasts (right). **(D)** Intersection of differentially expressed genes between invasive cytotrophoblasts and endovascular trophoblasts. **(E)** List of 12 shared differentially expressed genes.

The GSE93839 dataset was obtained from the GEO database to analyze gene expression in placental tissues from patients with PE compared to normal controls. Differential gene expression in invasive cytotrophoblasts and endovascular cytotrophoblasts was identified using the limma package (**Figure 1A**). GO analysis revealed that the monocyte activation pathway (GO:0030224) plays a significant role in the pathogenesis of PE, primarily involving monocyte differentiation and antigen presentation functions (**Figure 1B**). A PPI network was subsequently constructed using the STRING database to analyze interactions among the differentially expressed genes and identify core functional proteins (**Figure 1C**). Among the 12 shared DEGs identified in both invasive cytotrophoblasts and endovascular cytotrophoblasts, CD200 was selected as a candidate downregulated gene for downstream validation (**Figures 1D, E**). In addition, Spearman’s correlation analysis of the GSE93839 dataset revealed that CD200 was significantly positively correlated with the M2 macrophage marker MRC1 (ρ = 0.585, p < 0.001), whilst it was significantly negatively correlated with the immune activation-related marker CD86 (ρ = -0.416, p = 0.012), CD68 (ρ = -0.713, p < 0.001), NOS2 (ρ = -0.454, p = 0.030) and TNF (ρ = -0.475, p = 0.022) (**Supplementary Table 2**).

#### 3.3.2 Experimental detection of CD200 expression in placental tissue

**Figure 2:**
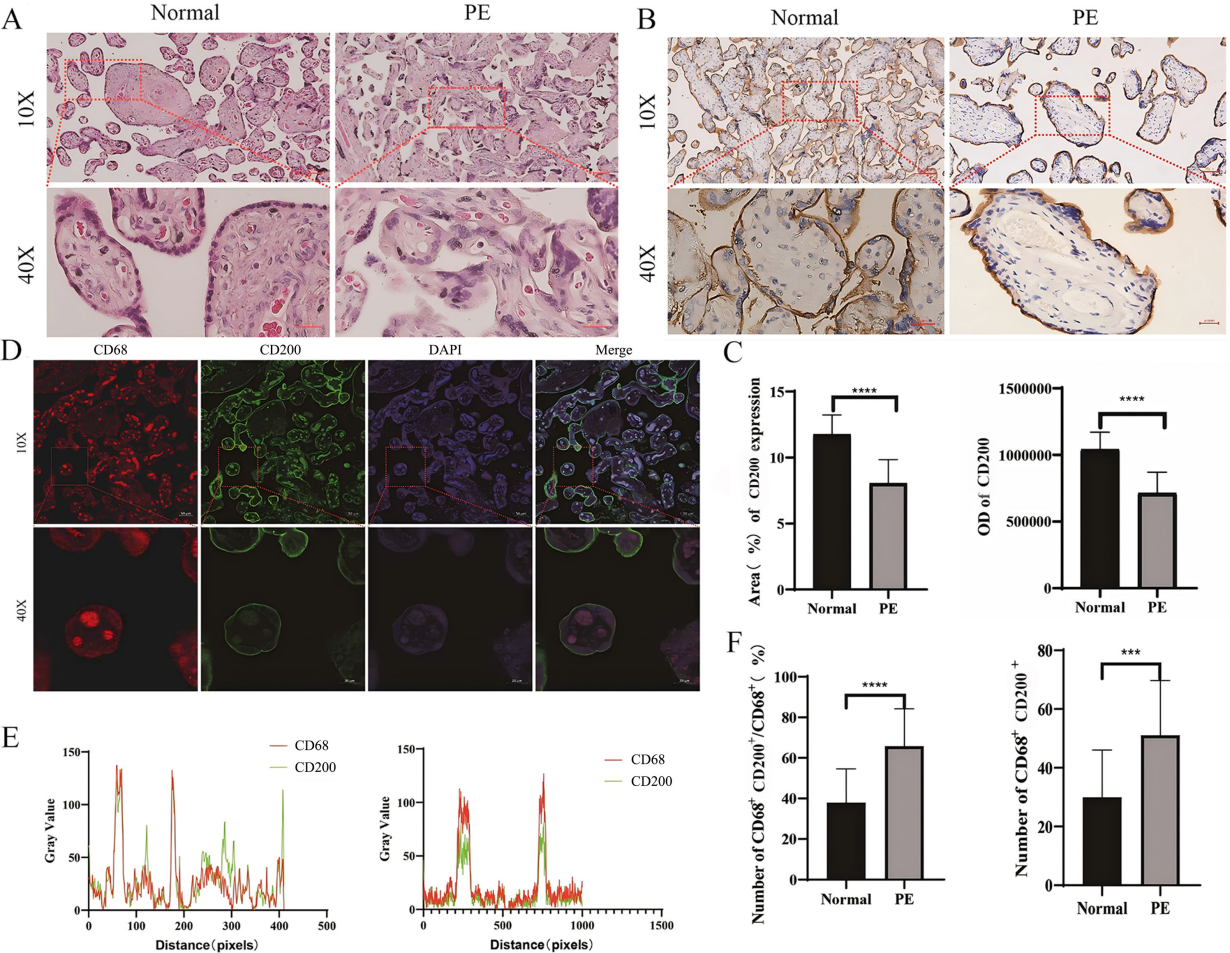
Detection of CD200 protein expression in placental tissues. **(A)** Hematoxylin and eosin (HE) staining of placental tissues from normal term pregnancy and PE under ×10 and ×40 magnification. **(B)** Immunohistochemical staining of placental tissues from normal term pregnancy and PE under ×10 and ×40 magnification. **(C)** Comparison of the CD200-positive area in placental tissues between normal term pregnancy and PE. **(D)** Immunofluorescence staining of placental tissues from PE. **(E)** Colocalization trend images under ×10 and ×40 magnification. **(F)** Comparison of CD200 expression between normal term pregnancy and PE placental tissues. ***p < 0.001, ****p < 0.0001.

HE staining results revealed that in normal term placentas, the villi exhibited abundant branching, uniform blood vessels, wide intervillous spaces, an intact trophoblast layer, and a smooth basement membrane. In contrast, preeclamptic placentas showed disorganized villous architecture, narrowed intervillous spaces, deposition of fibrinoid material, increased syncytial knots, and marked fibrosis. At 40× magnification, the normal placental villi showed a relatively regular structure with a homogeneous basement membrane, whereas the PE group exhibited a thickened basement membrane and swollen vascular endothelium, as shown in **Figure 2A**. IHC results demonstrated that the expression area and intensity of CD200 in preeclamptic placentas were significantly lower than those in normal term placentas (**Figures 2B, C**). Laser scanning confocal microscopy revealed that CD68 was primarily expressed in the cytoplasm, while CD200 was predominantly localized on the cell membrane, with partial cytoplasmic expression, suggesting co-localization (**Figure 2D**). The fluorescence intensity profile in **Figure 2E** confirmed a high degree of overlap between CD68 and CD200 signals in placental tissue, further validating their co-localization. IF double staining showed that the number and positive rate of CD68^+^CD200^+^ double-positive cells were significantly higher in preeclamptic placentas than the normal term group (both p < 0.001) (**Figure 2F**). Spatial co-localisation analysis indicates that CD200 signaling is primarily concentrated on the cell membrane and in the perimembrane region of CD68^+^ macrophages, suggesting a possible spatial association between CD200 and these cells.

#### 3.3.3 Altered expression of M1/M2 macrophage markers in placental tissues from PE

**Figure 3:**
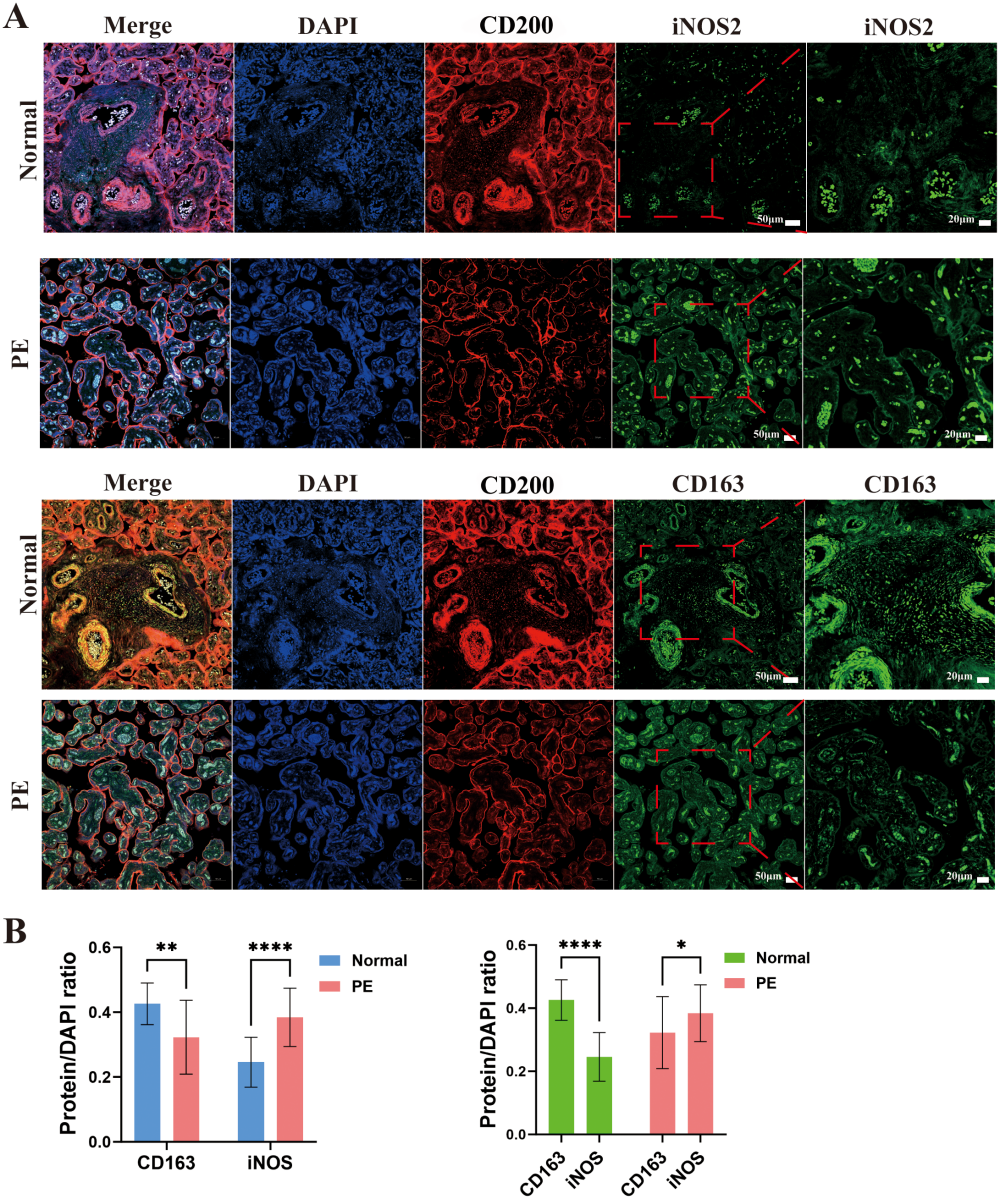
Detection of M1/M2 macrophage marker expression in placental tissues. **(A)** Immunofluorescence staining of serial placental sections from normal term pregnancy and PE showing the expression of the M1 macrophage marker iNOS and the M2 macrophage marker CD163. Compared with the normal term pregnancy group, placental tissues from the PE group showed increased iNOS expression and decreased CD163 expression. **(B)** Quantitative analysis of iNOS and CD163 expression normalized to DAPI in placentas from normal term pregnancy and PE. iNOS expression was significantly elevated in the PE group. ****p < 0.0001, **p < 0.01, * p < 0.05. When taking consecutive sections, photographs are taken of adjacent tissue areas, and their local features may exhibit a certain degree of similarity. Data are presented as mean ± SD. Each dot represents one individual sample or analyzed field, as applicable. Statistical comparisons were performed using Student’s t-test or Mann–Whitney U test according to data distribution. Protein-positive particle counts were normalized to the corresponding number of DAPI-stained nuclei in each field.

To further assess macrophage polarization in placental tissues, serial-section immunofluorescence staining was performed to detect the M1 macrophage marker iNOS and the M2 macrophage marker CD163 in the normal term pregnancy and PE groups. The use of serial sections allowed comparison of the expression patterns of different proteins in tissue regions with similar histological characteristics and corresponding anatomical localization. As shown in **Figure 3A**, compared with the normal term pregnancy group, placental tissues from the PE group exhibited increased expression of iNOS and decreased expression of CD163. Quantitative analysis after normalization to DAPI further confirmed that the expression levels of both iNOS and CD163 differed significantly between the two groups (all p < 0.05; **Figure 3B**). These findings suggest altered macrophage-associated inflammatory marker expression in PE placentas, characterized by increased iNOS-associated pro-inflammatory signals and reduced CD163-associated anti-inflammatory/tissue-remodeling signals.

### 3.4 Exploratory path analysis

To explore the potential pathways linking CD200 with PE, an exploratory analysis was conducted using candidate mediators that demonstrated significant associations with PE. These included TG, HDL, uric acid, albumin, creatinine, urea, ALT, AST, GGT, LDH, CK, and CK-MB. Preliminary weighted logistic regression and linear regression analyses confirmed that all these variables were significantly associated with both CD200 expression and the risk of PE.

**Figure 4:**
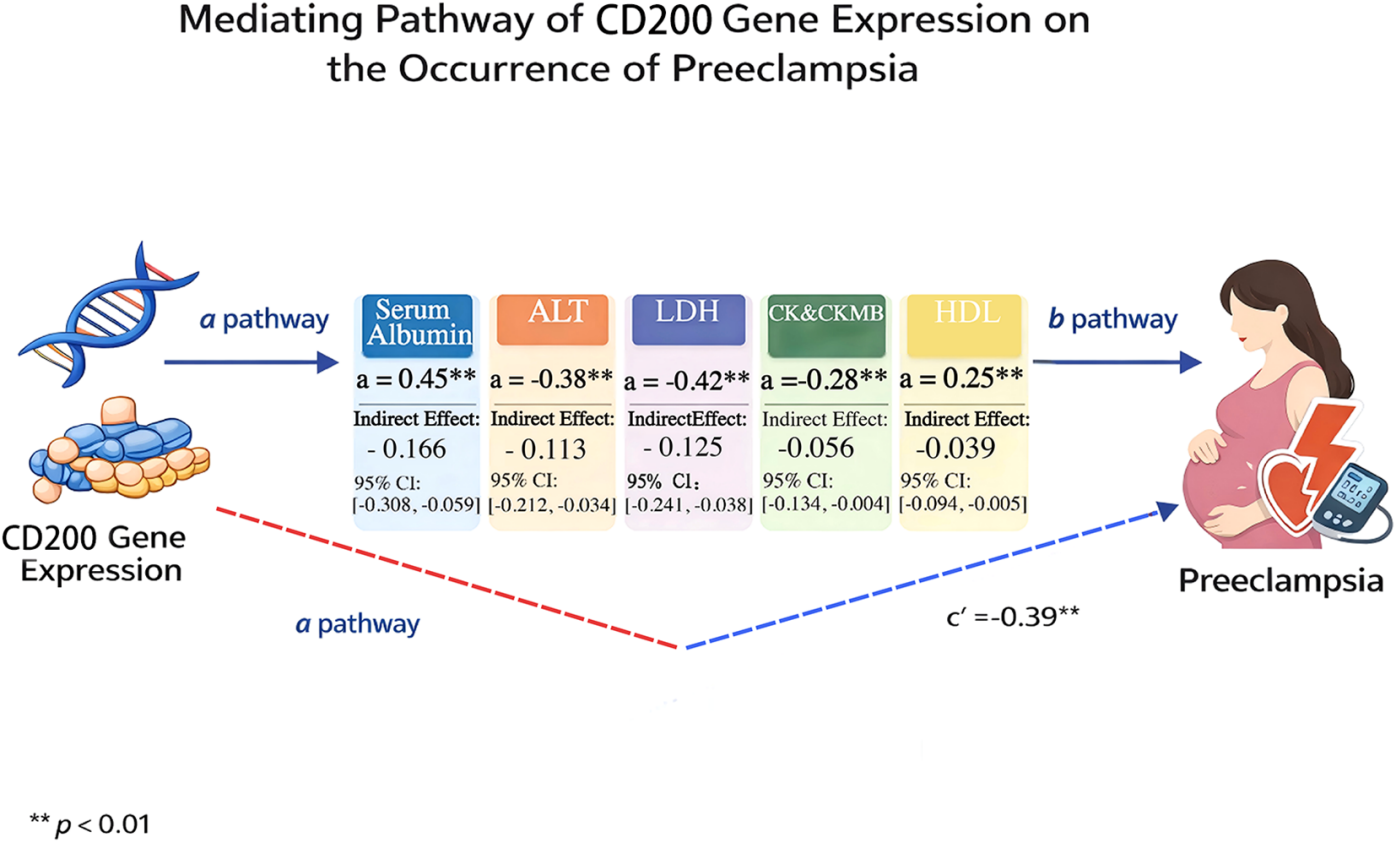
Exploratory path analysis of the associations between CD200 expression and PE through serum biomarkers. Models were adjusted for body mass index (BMI), systolic blood pressure, diastolic blood pressure, gestational age at delivery, mode of delivery, and neonatal birth weight. CI, confidence interval.

Among the tested serum biomarkers, HDL, serum albumin, ALT, CK, CK-MB, and LDH exhibited statistically significant effects on the association between CD200 expression and PE. As shown in **Figure 4**, increased CD200 expression was associated with elevated serum albumin (a = 0.45, p < 0.01) and elevated HDL (a = 0.25, p < 0.01), which in turn significantly reduced the risk of preeclampsia occurrence (indirect effects: −0.166 and −0.039, respectively). Furthermore, decreased CD200 expression was associated with increased levels of ALT, LDH, and CK/CK-MB, and these changes are also significantly associated with an increased risk of PE. **Supplementary Table 3** presents all candidate indicators identified prior to multiple-testing correction.

## 4 Discussion

CD200 is a key immunomodulatory molecule that plays a crucial role in maintaining immune tolerance at the maternal-fetal interface. Its deficiency may be associated with weakened inhibition of immune cells such as macrophages and enhanced local inflammatory responses, but the present data do not establish a direct causal or regulatory effect (10, 11). In this study, bioinformatics analysis identified CD200 as a differentially expressed gene, and GO analysis along with PPI network analysis suggested that it may represent a candidate immune-related molecule associated with PE rather than a key gene driving the occurrence and development of PE. To balance discovery potential against multiple-testing constraints in this modestly sized cohort, we prioritized raw *p*-values for candidate selection. All identified associations were interpreted conservatively and corroborated by independent histological and clinical assessments. In addition, the Spearman correlation analyses between CD200 and macrophage-related markers were based on transcriptomic data and therefore should be interpreted only as gene-expression-level associations. These correlations do not establish direct biological interactions between CD200 and macrophage markers, nor do they demonstrate macrophage polarization status within placental tissue. Further validation through molecular experiments and clinical cohort analysis confirmed that CD200 expression was significantly downregulated in the placental tissues of patients with PE, and its low expression was closely associated with placental villous structural damage, altered macrophage-related staining patterns, and adverse pregnancy outcomes. These findings are consistent with the established conclusion that the CD200-CD200R pathway is central to maintaining immune homeostasis and limiting excessive activation of myeloid cells (12). Taken together, our findings support an association between reduced placental CD200 expression and immune imbalance in PE, rather than proving that CD200 directly regulates macrophage polarization or drives systemic vascular injury.

Previous studies have demonstrated that placentas from preeclamptic pregnancies often exhibit ischemic and hypoxic changes, including villous hypoplasia, increased fibrinoid deposition, and villous vascular stenosis (13, 14). The observed villous structural disorganization and thickening of the basement membrane in the HE staining results of this study are highly consistent with these pathological features, suggesting that the placenta is in a state of chronic hypoperfusion and oxidative stress. IHC results revealed that both the CD200-positive area and the comprehensive CD200 expression index were significantly decreased in preeclamptic placentas (p < 0.001). Previous animal model studies have indicated that CD200 deficiency can lead to sustained macrophage activation and excessive production of pro-inflammatory cytokines (14). Related research also suggests that CD200 expression in normal pregnancy placentas contributes to maintaining maternal-fetal immune tolerance, and its downregulation may be associated with an enhanced inflammatory bias (15). Therefore, the observed downregulation of CD200 in this study suggests a weakening of negative immune regulatory signals at the maternal-fetal interface, potentially creating conditions for macrophage functional imbalance. However, this statement should be understood as an association-based interpretation rather than evidence that CD200 directly influences macrophage-mediated immune imbalance. IF further confirmed that CD200 is primarily localized to the membrane and perimembrane regions of CD68^+^ macrophages, and the proportion of CD68^+^ CD200^+^ double-positive cells was increased in the PE group. Notably, immunofluorescence analysis showed an increased proportion of CD68^+^CD200^+^ cells, while immunohistochemistry indicated an overall decrease in CD200 expression levels. These findings are not necessarily contradictory. Increased macrophage infiltration in preeclamptic placentas has been confirmed by multiple studies (16, 17). Under such circumstances, even if the CD200 expression intensity per cell decreases, the proportion of double-positive cells may still rise due to the increased number of macrophages. However, because we did not perform compartment-specific quantification of CD68^+^ macrophage density, CD200 intensity per CD68^+^cell, or CD200 expression in CD68-negative regions, this interpretation should be regarded as cautious and inferential rather than definitive. More importantly, the CD200 signal was concentrated in the cell membrane region, consistent with the biological characteristics of CD200 as a membrane-bound immunoregulatory molecule. In addition, while our staining suggests a macrophage-associated localization of CD200, the current panel lacked markers specific to trophoblasts, endothelial cells, and stromal compartments. Consequently, the precise cellular and compartmental origins of CD200 downregulation remain unresolved. Previous research on pregnancy immune tolerance has shown that CD200 expression by trophoblasts or related cells helps suppress local Th1-type inflammatory responses and maintain immune balance at the maternal-fetal interface (15). Consequently, the findings of this study are consistent with the concept that reduced CD200 may be associated with impaired maternal-fetal immune tolerance and a more inflammatory immune environment; however, this remains an association-based interpretation and does not establish *in vivo* causality. Consistent with this interpretation, serial-section immunofluorescence further showed increased iNOS expression and decreased CD163 expression in PE placentas, suggesting changes in macrophage-associated inflammatory marker patterns (18, 19). In the present study, iNOS and CD163 were used only as representative macrophage-associated inflammatory markers, rather than as definitive indicators of discrete M1 or M2 polarization states. These observations indicate marker-associated pro-inflammatory and anti-inflammatory/regulatory shifts, but they should not be interpreted as evidence of fixed or binary macrophage polarization. It should be noted that placental macrophage phenotypes exist along a functional spectrum and cannot be fully classified based on a limited marker panel alone. Therefore, the terms “M1-like” and “M2-like,” if used, should be understood as simplified descriptions of marker-associated tendencies rather than distinct macrophage subsets. Accordingly, the observed increase in iNOS and decrease in CD163 should be interpreted as evidence of altered macrophage-associated marker expression within the placental microenvironment, rather than proof of a transition between two discrete macrophage polarization states. Because serial sections allow comparison of different proteins in tissue regions with similar histological characteristics and corresponding anatomical localization, these findings provide additional spatially comparable evidence for altered macrophage marker-associated patterns in PE. Overall, these tissue-level observations support the view that reduced placental CD200 expression is associated with a more inflammatory local immune environment in PE; however, they remain descriptive and do not directly demonstrate that CD200 reduction is the cause of macrophage infiltration or macrophage phenotypic changes. Therefore, any interpretation linking CD200 downregulation to macrophage functional alterations should be regarded as hypothesis-generating and requires validation using broader immune-marker panels, single-cell approaches, and functional experiments.

It is also important to distinguish CD200 from classical oxidative stress pathways in PE. PE is widely recognized as a disorder involving abnormal placentation, endothelial dysfunction, inflammation, and oxidative stress (20, 21). In this broader context, CD200 should not be considered equivalent to HMOX1/HO-1 or interpreted as a novel oxidative stress pathway per se. Unlike HMOX1, which functions primarily as an antioxidant enzyme involved in heme degradation and cytoprotection, CD200 is an immune checkpoint molecule whose principal known role is to restrain myeloid-cell activation through CD200R1-mediated signaling (12, 22). Therefore, our findings more likely place CD200 within the broader network of inflammatory and redox dysregulation in PE, as a potential immunoregulatory component, rather than as an isolated upstream redox regulator.

The results of the exploratory path analysis suggest that reduced placental CD200 expression is statistically associated with systemic patterns characteristic of PE, in a manner consistent with the conceptual sequence “macrophage immune regulation - inflammation amplification - endothelial dysfunction” (23, 24). HDL, albumin, ALT, LDH, CK, and CK-MB showed significant indirect associations in the model. These findings indicate that placental CD200 downregulation may be linked to concurrent alterations in metabolic, endothelial permeability-related, and organ injury-related clinical indicators in PE (25). HDL is commonly regarded as a protective lipid-related marker with anti-inflammatory and endothelial regulatory properties, whereas reduced albumin levels in PE may reflect endothelial permeability changes and altered systemic metabolic or nutritional status (26, 27). Reduced CD200 expression was statistically associated with altered HDL and albumin levels and an inflammatory/injury-related biomarker profile in PE; however, causal regulatory mechanisms were not established in this study. Similarly, ALT, LDH, CK, and CK-MB, which are clinically used indicators related to hepatic injury, tissue hypoxia or cellular injury, and myocardial stress, respectively, also showed significant indirect associations (28). LDH, as an indicator of cellular hypoxia and tissue injury, shows a positive correlation between its elevation and disease severity (29). Abnormalities in CK and CK-MB suggest a state of myocardial stress against a background of increased circulatory load. These results suggest that reduced placental CD200 expression covaries with a broader PE-associated clinical phenotype involving metabolic imbalance and organ injury-related biomarkers. However, the associations involving HDL, albumin, ALT, LDH, CK, and CK-MB should be regarded as exploratory statistical relationships and should not be interpreted as evidence of CD200-related mechanistic pathways. Although this study did not observe a direct mediating effect of renal function indicators such as creatinine and urea with CD200, previous literature has confirmed that PE is often accompanied by glomerular endothelial injury and abnormal uric acid metabolism (30, 31). However, because placental CD200 expression, serum biomarkers, and PE status was all measured at or near delivery, these findings should be interpreted as hypothesis-generating rather than evidence supporting a causal pathway. Thus, the path analysis should be interpreted as describing statistical associations rather than demonstrating a CD200-mediated biological pathway.

This study integrated clinical sample analysis, bioinformatic screening, and histological experimental validation, revealing the potential association between downregulated CD200 expression and PE-related placental immune alterations and multi-organ injury-related clinical features, thereby providing a candidate placental molecule associated with immune alterations in PE. However, this study has several key limitations that require clarification. First, the study lacks functional validation. Relying solely on clinical samples and bioinformatics analyses can only confirm a correlation between downregulated CD200 expression and PE, but cannot establish a causal relationship. Further *in vivo* and *in vitro* experiments are necessary to determine whether downregulated CD200 expression is a contributing factor to PE-related immune dysregulation or a subsequent alteration following disease progression, and to elucidate the downstream molecular pathways through which it may influence macrophages. Therefore, the present study cannot determine whether CD200 has a direct regulatory effect on macrophages or other placental cell populations. Second, unmeasured clinical confounding variables may have influenced the results. This study is a single-center retrospective analysis with a limited sample size (n = 106), which is prone to selection bias and restricts the generalizability of the findings. In particular, substantial differences between the PE and control groups in gestational age at delivery, mode of delivery, and neonatal birth weight may themselves influence placental histology, immune-cell distribution, and gene/protein expression. Moreover, because gestational-age-matched preterm non-PE controls were not included, reduced placental CD200 expression cannot be attributed specifically to PE without caution. Third, the cross-sectional study design precludes temporal inference. Because placental tissues were collected only at the time of delivery, it is impossible to determine whether downregulated CD200 expression precedes immune dysregulation events, such as altered macrophage marker-associated patterns, or occurs secondary to PE progression. This limitation hampers the assessment of the early predictive value of CD200. Fourth, the immune evidence in the present study remains incomplete. We did not perform CD200R1 staining, placental inflammatory cytokine profiling, or functional trophoblast-macrophage experiments with CD200 perturbation. In addition, although iNOS and CD163 are commonly used polarization-related markers, they do not fully capture the continuum and complexity of placental macrophage states. Thus, iNOS and CD163 should be regarded as limited marker readouts reflecting inflammatory or regulatory tendencies, rather than definitive markers sufficient to assign placental macrophages to M1 or M2 states. Therefore, the current findings should be interpreted as changes in macrophage-associated marker expression rather than definitive evidence of fixed M1/M2 polarization states. Future research should adopt a prospective, multicenter, longitudinal cohort design, with serial sampling throughout gestation to track the expression dynamics of CD200. Concurrent *in vivo* and *in vitro* functional experiments will be essential for establishing causality and elucidating the underlying molecular regulatory mechanisms. Incorporating single-cell sequencing technology could further characterize the expression profile of CD200 across specific placental cell subpopulations, thereby enhancing its potential for clinical translation. In particular, single-cell transcriptomic or spatial multi-omics analyses combined with expanded macrophage marker panels would be helpful to define placental macrophage states more accurately without relying solely on the simplified M1/M2 framework.

Fifth, our findings were derived from bulk transcriptomic screening and tissue-level staining. These approaches cannot resolve cell-intrinsic functional interactions or compartment-specific regulatory events at the single-cell level, and tissue-level protein readouts may partially reflect changes in cellular composition rather than direct regulatory effects within specific cell types. Similarly, bulk transcriptomic data cannot distinguish whether the observed CD200-related signal originates from trophoblasts, macrophages, endothelial cells, stromal cells, or changes in the relative abundance of these cell populations. Therefore, the present study cannot infer cell-specific ligand-receptor interactions or dynamic intercellular communication at the maternal-fetal interface.

## 5 Conclusion

This study demonstrates that CD200 expression is significantly downregulated in placental tissues from patients with PE, and its low expression is closely associated with placental structural damage and altered macrophage-associated spatial and marker-expression patterns. Exploratory Path Analysis suggests potential statistical associations between CD200 downregulation and metabolic- and organ injury-related biomarker profiles. These associations, including those involving HDL, albumin, ALT, LDH, CK, and CK-MB, should be interpreted as exploratory statistical findings rather than evidence of mechanistic pathways. These findings identify altered placental CD200 expression as a candidate molecule associated with immune alterations in PE. However, the observed changes in iNOS and CD163 expression should be interpreted as macrophage-associated inflammatory marker alterations rather than definitive evidence of discrete M1/M2 polarization states. Placental macrophages likely exist along a phenotypic continuum, and the current marker panel is insufficient to define their full functional status. Further cell-specific research is required to identify the specific tissue compartments in the placenta responsible for alterations in CD200 expression, and mechanistic studies are needed to elucidate their role. Given the observational nature of this study, the present findings should be interpreted as associations and do not demonstrate that CD200 regulates macrophage function or causally contributes to PE progression.

## Data Availability

The raw data supporting the conclusions of this article will be made available by the authors, without undue reservation.

## References

[1] Dimitriadis E Rolnik DL Zhou W Estrada-Gutierrez G Koga K Francisco RPV . Pre-eclampsia. Nat Rev Dis Primers. (2023) 9:8. doi: 10.1038/s41572-023-00417-6 36797292

[2] Erez O Romero R Jung E Chaemsaithong P Bosco M Suksai M . Preeclampsia/eclampsia: the conceptual evolution of a syndrome. Am J Obstet Gynecol. (2022) 226:S786–803. doi: 10.1016/j.ajog.2021.12.001 35177220 PMC8941666

[3] Melchiorre K Giorgione V Thilaganathan B . The placenta and preeclampsia: villain or victim? Am J Obstet Gynecol. (2022) 226:S954–62. doi: 10.1016/j.ajog.2020.10.024 33771361

[4] Li J Wang Z Qin X Zhong M-C Tang Z Qian J . CD200R1-CD200 checkpoint inhibits phagocytosis differently from SIRPα-CD47 to suppress tumor growth. Nat Commun. (2025) 16:5145. doi: 10.1038/s41467-025-60456-3 40461553 PMC12134331

[5] Liu J-Q Hu A Zhu J Yu J Talebian F Bai X-F . CD200-CD200R pathway in the regulation of tumor immune microenvironment and immunotherapy. Adv Exp Med Biol. (2020) 1223:155–65. doi: 10.1007/978-3-030-35582-1_8 32030689 PMC7339106

[6] Hayakawa K Wang X Lo EH . CD200 increases alternatively activated macrophages through cAMP-response element binding protein - C/EBP-beta signaling. J Neurochem. (2016) 136:901–6. doi: 10.1111/jnc.13492 26670206 PMC4755817

[7] Denieffe S Kelly RJ McDonald C Lyons A Lynch MA . Classical activation of microglia in CD200-deficient mice is a consequence of blood brain barrier permeability and infiltration of peripheral cells. Brain Behav Immun. (2013) 34:86–97. doi: 10.1016/j.bbi.2013.07.174 23916893

[8] George EM Granger JP . Heme oxygenase in pregnancy and preeclampsia. Curr Opin Nephrol Hypertens. (2013) 22:156–62. doi: 10.1097/MNH.0b013e32835d19f7 23328500 PMC3829378

[9] Clark DA Arredondo JL Dhesy-Thind S . The CD200 tolerance-signaling molecule and its receptor, CD200R1, are expressed in human placental villus trophoblast and in peri-implant decidua by 5 weeks’ gestation. J Reprod Immunol. (2015) 112:21–3. doi: 10.1016/j.jri.2015.05.005 26123445

[10] Ding J Zhang Y Cai X Diao L Yang C Yang J . Crosstalk between trophoblast and macrophage at the maternal-fetal interface: current status and future perspectives. Front Immunol. (2021) 12:758281. doi: 10.3389/fimmu.2021.758281 34745133 PMC8566971

[11] Hoek RM Ruuls SR Murphy CA Wright GJ Goddard R Zurawski SM . Down-regulation of the macrophage lineage through interaction with OX2 (CD200). Science. (2000) 290:1768–71. doi: 10.1126/science.290.5497.1768 11099416

[12] Kotwica-Mojzych K Jodłowska-Jędrych B Mojzych M . CD200:CD200R interactions and their importance in immunoregulation. Int J Mol Sci. (2021) 22:1602. doi: 10.3390/ijms22041602 33562512 PMC7915401

[13] Wang S Hu Q Liao H Wang K Yu H . Perinatal outcomes of pregnancy in women with scarred uteri. IJWH. (2023) 15:1453–65. doi: 10.2147/IJWH.S422187 37746587 PMC10517689

[14] Franz S Muñoz LE Heyder P Herrmann M Schiller M . Unconventional apoptosis of polymorphonuclear neutrophils (PMN): staurosporine delays exposure of phosphatidylserine and prevents phagocytosis by MΦ-2 macrophages of PMN. Clin Exp Immunol. (2015) 179:75–84. doi: 10.1111/cei.12412 24995908 PMC4260899

[15] Clark DA Keil A Chen Z Markert U Manuel J Gorczynski RM . Placental trophoblast from successful human pregnancies expresses the tolerance signaling molecule, CD200 (OX-2)*. Am J Reprod Immunol. (2003) 50:187–95. doi: 10.1034/j.1600-0897.2003.00086.x 14629022

[16] Hu P Luo S Qu G Luo Q Tian Y Huang K . Identification and validation of feature genes associated with M1 macrophages in preeclampsia. Aging (Albany NY). (2023) 15:13822–39. doi: 10.18632/aging.205264 38048229 PMC10756132

[17] Faas MM De Vos P . Innate immune cells in the placental bed in healthy pregnancy and preeclampsia. Placenta. (2018) 69:125–33. doi: 10.1016/j.placenta.2018.04.012 29748088

[18] Vilotić A Nacka-Aleksić M Pirković A Bojić-Trbojević Ž Dekanski D Jovanović Krivokuća M . IL-6 and IL-8: an overview of their roles in healthy and pathological pregnancies. Int J Mol Sci. (2022) 23:14574. doi: 10.3390/ijms232314574 36498901 PMC9738067

[19] Li R Huang Z Kong L Cai W Li X Hsieh C . CAT/SOD-enriched achyranthes bidentata nanovesicles mitigate TMJOA via ROS scavenging and JNK/FOXO1 pathway inhibition. J Nanobiotechnol. (2025) 23:782. doi: 10.1186/s12951-025-03834-9 41430692 PMC12723957

[20] Wu X Li X Wu Y Yang H Wu J He L . Comprehensive identification of immune-related biomarkers and therapeutic targets in preeclampsia: integrative bioinformatics and experimental validation. BMC Pregnancy Childbirth. (2025) 25:1007. doi: 10.1186/s12884-025-08169-9 41044520 PMC12495723

[21] Torres-Torres J Espino-Y-Sosa S Martinez-Portilla R Borboa-Olivares H Estrada-Gutierrez G Acevedo-Gallegos S . A narrative review on the pathophysiology of preeclampsia. Int J Mol Sci. (2024) 25:7569. doi: 10.3390/ijms25147569 39062815 PMC11277207

[22] Jiang J Xu Y Chen D Li J Zhu X Pan J . Pan-cancer analysis of immune checkpoint receptors and ligands in various cells in the tumor immune microenvironment. Aging (Milano). (2024) 16:11683–728. doi: 10.18632/aging.206053 39120585 PMC11346784

[23] Bhat PV Vinod V Priyanka AN Kamath A . Maternal serum lipid levels, oxidative stress and antioxidant activity in pre-eclampsia patients from southwest India. Pregnancy Hypertension. (2019) 15:131–3. doi: 10.1016/j.preghy.2018.12.010 30825910

[24] Fernando M Ellery SJ Marquina C Lim S Naderpoor N Mousa A . Vitamin D-binding protein in pregnancy and reproductive health. Nutrients. (2020) 12:1489. doi: 10.3390/nu12051489 32443760 PMC7285222

[25] Phipps EA Thadhani R Benzing T Karumanchi SA . Pre-eclampsia: pathogenesis, novel diagnostics and therapies. Nat Rev Nephrol. (2019) 15:275–89. doi: 10.1038/s41581-019-0119-6 30792480 PMC6472952

[26] Einbinder Y Biron-Shental T Agassi-Zaitler M Tzadikevitch-Geffen K Vaya J Khatib S . High-density lipoproteins (HDL) composition and function in preeclampsia. Arch Gynecol Obstet. (2018) 298:405–13. doi: 10.1007/s00404-018-4824-3 29938347

[27] Saitou T Watanabe K Kinoshita H Iwasaki A Owaki Y Matsushita H . Hypoalbuminemia is related to endothelial dysfunction resulting from oxidative stress in parturients with preeclampsia. Nagoya J Med Sci. (2021) 83:741–8. doi: 10.18999/nagjms.83.4.741 34916718 PMC8648536

[28] Hammoud GM Ibdah JA . Preeclampsia-induced liver dysfunction, HELLP syndrome, and acute fatty liver of pregnancy. Clin Liver Dis. (2014) 4:69–73. doi: 10.1002/cld.409 30992924 PMC6448736

[29] Khidri FF Shaikh F Khowaja I-U-H Riaz H . Role of lactate dehydrogenase in the prediction of severity in pre- eclampsia. Curr Hypertens Rev. (2020) 16:223–8. doi: 10.2174/1573402116666200720001032 32684152

[30] Yang C Baker PN Granger JP Davidge ST Tong C . Long-term impacts of preeclampsia on the cardiovascular system of mother and offspring. Hypertension. (2023) 80:1821–33. doi: 10.1161/HYPERTENSIONAHA.123.21061 37377011

[31] Deer E Herrock O Campbell N Cornelius D Fitzgerald S Amaral LM . The role of immune cells and mediators in preeclampsia. Nat Rev Nephrol. (2023) 19:257–70. doi: 10.1038/s41581-022-00670-0 36635411 PMC10038936

