# Supplementary material for: Downregulation of CD200 in the placenta of preeclampsia: a potential regulator of macrophage-mediated immune imbalance at the maternal-fetal interface
Source: Front Immunol. 2026 Jun 30;17:1830162. doi: 10.3389/fimmu.2026.1830162 (PMC13364593; doi:10.3389/fimmu.2026.1830162)
Supplement: Supplementary file 1 [file DataSheet1.pdf]

Table S1. Perinatal management and foetal complications associated with hypertensive disorders of pregnancy

Characteristics	Statistics	Values(n, %)
**Antenatal magnesium sulfate**		
	yes	30（0.53）
	no	27（0.47）
**Antenatal corticosteroids**		
	yes	17（0.30）
	no	40（0.70）
**Antenatal antihypertensive treatment**		
	yes	31（0.54）
	no	26（0.46）
**Onset of disease**		
	yes	29（0.51）
	no	28（0.49）
**PE Severity**		
	yes	40（0.70）
	no	17（0.30）
**Fetal growth restriction**		
	yes	30（0.53）
	no	27（0.47）

Table S2. Analysis of the correlation between CD200 and M1/M2 macrophage markers using the GSE93839 dataset

Gene	Spearman_rho	P_value	FDR
**MRC1**	0.58517844345799465	0.00017846890346768	0.001068579864236
**CD86**	-0.415778937	0.01168746579774264	0.0350612345756942
**CX3CR1**	-0.163577964	0.45579456667268525	0.5259168076992522
**CD163**	-0.279644269	0.19625242340232063	0.3270873723372011
**IL10**	-0.328638508	0.12574670695129	0.314366767378225
**ARG1**	-0.40513834	0.0551386874533403	0.1654160623600209
**NOS2**	-0.453557312	0.029727123393078658	0.1486356169653933
**TNF**	-0.475296443	0.021901740423313585	0.0448635616965393 3
**CD68**	-0.713438735	0.0001324270913415873 5	0.0019864063701238 105

Table S3. Candidate indicators prior to correction for multiple tests in exploratory path analysis

Mediator (M)	Path a Coefficient ($X \rightarrow M$)	Indirect Effect ($a \times b$)	95% Bootstrap CI	Significance
**Serum Albumin**	0.45**	-0.166	[-0.274, -0.085]	**
**platelet**	0.13*	-0.021	[-0.039, -0.002]	*
**LDH**	-0.42**	-0.125	[-0.213, -0.059]	**
**CK & CKMB**	-0.28**	-0.056	[-0.115, -0.016]	**
**WBC**	-0.19*	-0.047	[-0.094, -0.004]	*
**ALT**	-0.38**	-0.113	[-0.188, -0.053]	**
**HDL**	0.25**	-0.039	[-0.081, -0.013]	**

**** indicates $p < 0.01$, *$p < 0.05$**
